# Supplementary material for: Large fault slip peaking at trench in the 2011 Tohoku-oki earthquake
Source: Nat Commun. 2017 Jan 11;8:14044. doi: 10.1038/ncomms14044 (PMC5241695; doi:10.1038/ncomms14044)
Supplement: Supplementary Information — Supplementary Figures, Supplementary Tables and Supplementary References. [file ncomms14044-s1.pdf]

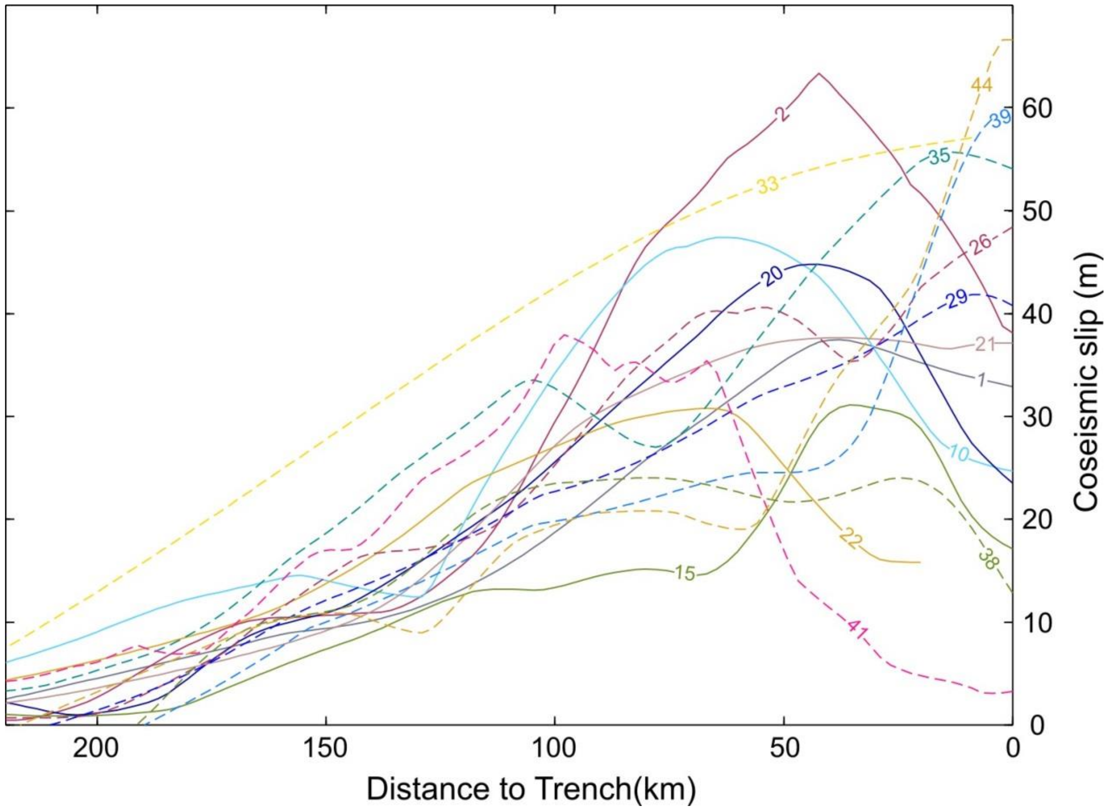

Supplementary Figure 1 | Published rupture models of the Tohoku-oki earthquake that included tsunami data as constraints. Each curve is labeled with its model number as in Supplementary Tables 1 and 2. This is a subset of the models shown in Fig. 1. The use of tsunami data in some of the recent models helped improve near-trench resolution of slip models such as models 26 and 35, but not in all the recent models.

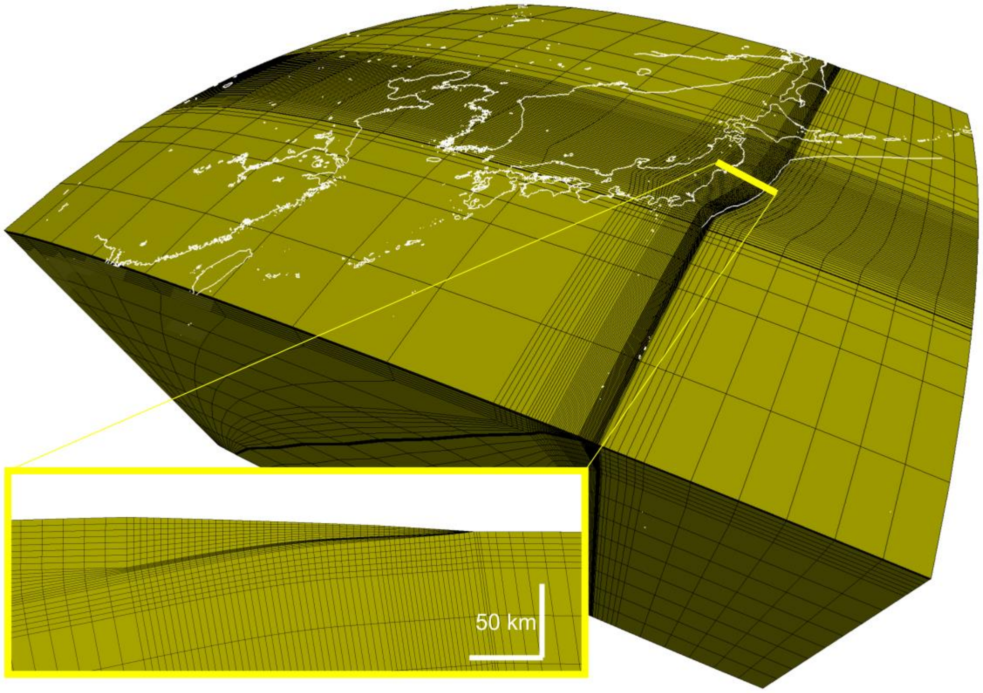

Supplementary Figure 2 | Finite element mesh used in this work for modeling coseismic deformation of the Tohoku-oki earthquake. Inset: cross-section view of the mesh along the central corridor for SDB calculation.

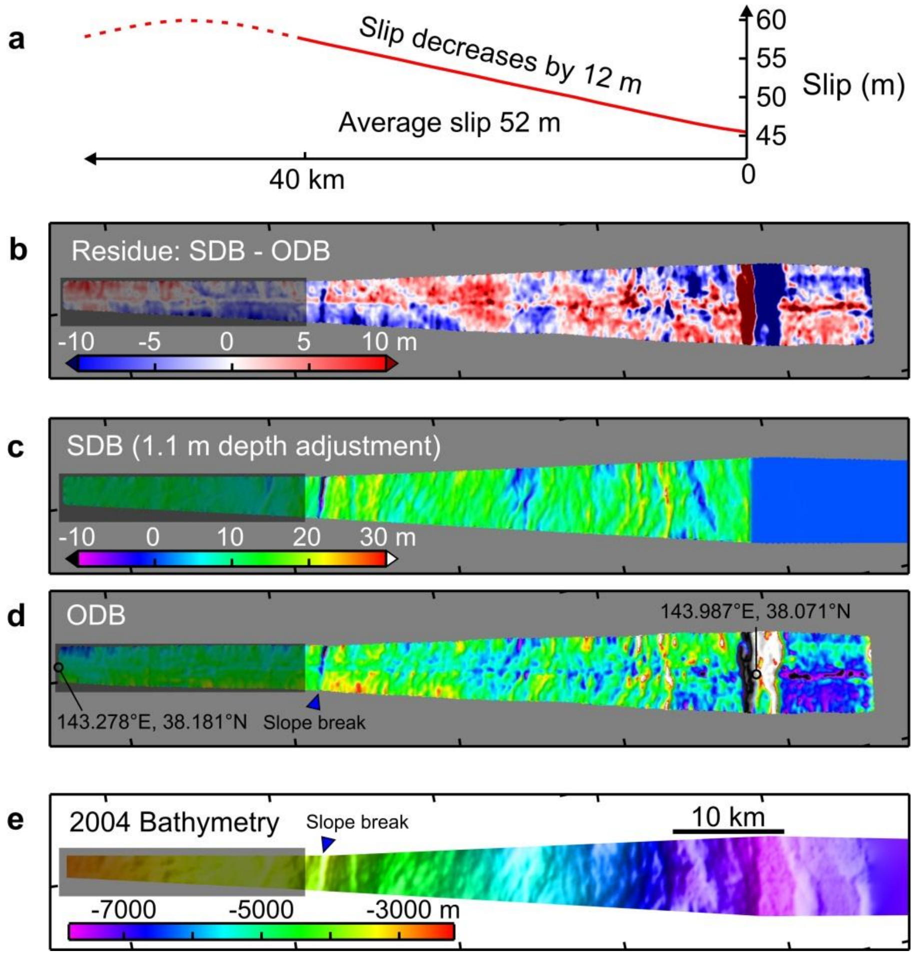

Supplementary Figure 3 | Optimal SDB model for the central corridor using 2004–2011 ODB. **a**, Fault slip distribution over the most seaward 40 km. **b**, Residue between the SDB and 2004-2011 ODB. **c**, SDB produced using the slip distribution shown in **a**. **d**, 2004–2011 ODB. The limited coverage seaward of the trench renders the OBD image much less reliable than the 1999–2011 image (Fig. 3d). **e**, Bathymetry acquired in 2004.

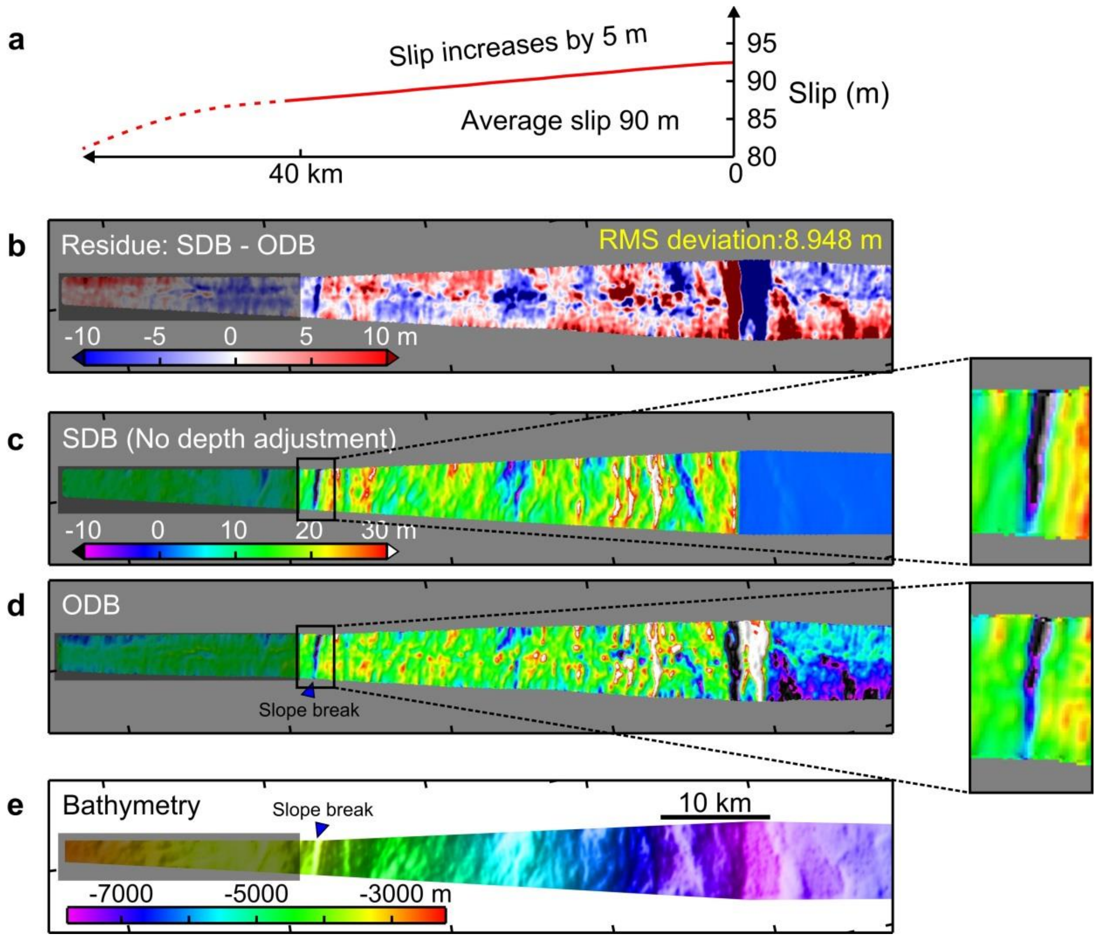

Supplementary Figure 4 | SDB model for the central corridor with an average fault slip of 90 m and zero optimal depth adjustment. Otherwise the figure is similar to Fig. 3. **a**, Fault slip distribution over the most seaward 40 km. **b**, Residue between the SDB and 1999-2011 ODB. **c**, SDB produced using the slip distribution shown in **a**. **d**, 1999-2011 ODB. **e**, Bathymetry acquired in 1999. The zoom-in area in **c** and **d** shows that the SDB incorrectly predicts a wider area of local bathymetry decrease than in the ODB.

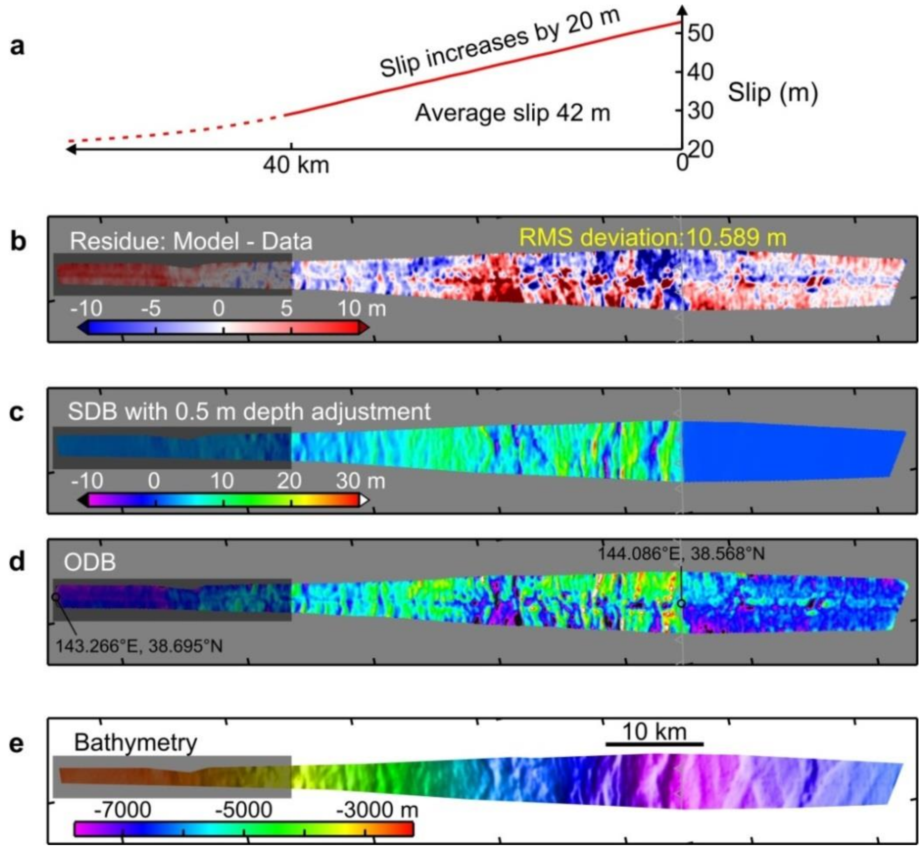

Supplementary Figure 5 | Optimal SDB model along bathymetry track MY101 about 50 km north of the central corridor. Otherwise the figure is similar to Fig. 3. **a,** Fault slip distribution. **b**, Residue between the SDB and ODB. **c**, SDB produced using the slip distribution shown in **a**. **d,** ODB from data collected in 1999 and May 2011. **e**, Bathymetry acquired in 1999.

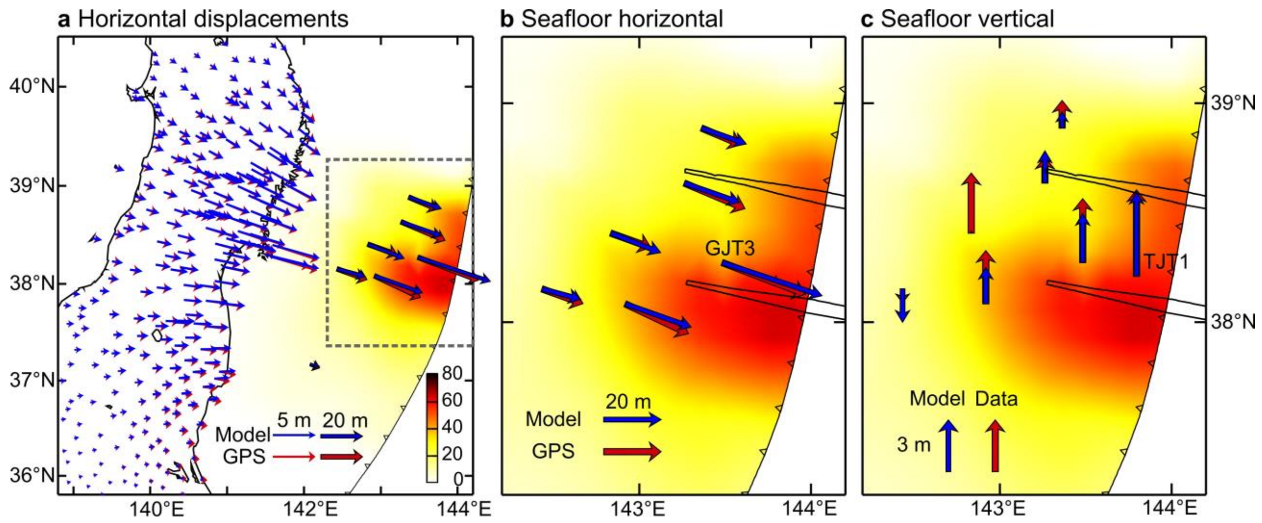

Supplementary Figure 6 | A slip distribution of the Tohoku-oki earthquake that can satisfy differential bathymetry and are also compatible with other geodetic data. a, Broad-scale view of the model slip distribution (in meters) and model-predicted horizontal displacements in comparison with land-based[1] and seafloor[2,3] GPS measurements. **b**, Enlarged view of the main rupture area (dashed box in **a**) with the two bathymetry tracks shown. **c**, View of the main rupture area showing model-predicted uplift in comparison with coseismic uplift inferred from seafloor GPS[2,3] or OBP[4] data. The slip model shown in this figure represents an earthquake of M_w=9.02 if rigidity is assumed to be 40 GPa. The slip distribution is not obtained by inversion but is based on hand-extrapolating the slip distribution shown in Fig. 3a and Supplementary Fig. 5a. The forward modeling of surface displacements is done with the same mesh as shown in Supplementary Fig. 2. The purpose is not to fit all the geodetic data, but to show that the magnitude of seafloor displacements is consistent with most data, especially the ODB data at site TJT1. A more complete understanding of the heterogeneous shallow slip distribution would require more near-trench observations.

Supplementary Table 1 | Rupture models of the Tohoku-oki earthquake obtained by including seafloor GPS data

Model No.	Reference (Ref No.)	Data used	Seafloor GPS sites used	Peak slip* (m)	Peak slip along the corridor (m)
1	Gusman et al., 2012 (5)	Tsunami (seafloor pressure and tide gauge), land and seafloor GPS	5 sites	42.0	37.5
2	Hooper et al., 2013 (6)	Land and seafloor GPS, tsunami (seafloor pressure gauges), satellite altimetry	5 sites	78.7	63.4
3	Iinuma et al., 2012 (7)	Land and seafloor GPS, seafloor pressure sensors	7 sites	87.9	83.0
4	Imakiire and Koarai, 2012 (8)	Land and seafloor GPS	≤5 sites	59.2	54.1
5	T. Ito et al., 2011 (9)	Land and seafloor GPS	3 sites	59.8	31.0
6	Y. Ito et al., 2011 (4)	Seafloor pressure and acoustic ranging records	1 site	80.0	80.0
7	Kubo and Kakehi, 2013 (10)	Teleseismic body waves, land and seafloor GPS	5 sites	42.9	39.9
8	Kyriakopoulos et al., 2013 (11)	Land and seafloor GPS (FEM inversion)	5 sites	39.2	35.9
9	Lee et al., 2011 (12)	Teleseismic waves, land and seafloor GPS, strong motion	5 sites	56.1	52.0
10	Minson et al., 2014 (13)	High-rate GPS, land and seafloor GPS, tsunami	7 sites	73.8	47.4
11	Ozawa et al., 2012 (1)	Land and seafloor GPS	7 sites	64.0	48.0
12	Perfettini and Avouac, 2014 (14)	Land and seafloor GPS	6 sites	53.6	49.2
13	Pollitz et al., 2011 (15)	Land (including very far field) and seafloor GPS	5 sites	38.5	35.5
14	Pulvirenti et al., 2014 (16)	Land and seafloor GPS	5 sites	35.1	30.3
15	Romano et al., 2014 (17)	Land and seafloor GPS, tsunami (DART, coastal wave, seafloor pressure gauges) (FEM inversion)	7 sites	44.7	31.1

16	Shao et al., 2012 (18)	Teleseismic, local strong motion, land & seafloor GPS	5 sites	63.5	60.5
17	Silverii et al., 2014 (19)	Land and seafloor GPS	7 sites	57.7	49.0
18	Wang et al., 2012 (20)	Land and seafloor GPS, InSAR	5 sites	49.9	48.7
19	Wang et al., 2013 (21)	Strong motion (K & KiK nets), land and seafloor GPS	5 sites	47.6	46.5
20	Wei et al., 2012 (22)	Strong motion, land and seafloor GPS, DART data	5 sites	48.0	44.8
21	Wei et al., 2014 (23)	Tsunami (open ocean GPS buoy), land and seafloor GPS	5 sites	48.7	37.7
22	Yokota et al., 2011 (24)	Strong motion, teleseismic, land and seafloor GPS, tsunami	5 sites	35.3	30.8
23	Yue and Lay, 2013 (25)**	High-rate GPS, teleseismic P wave, Rayleigh wave, seafloor GPS	5 sites	70.4	41.7
24	Zhou et al., 2014 (26)	Land and seafloor GPS	7 sites	53.0	46.5

* The peak values were obtained from the original finite fault slip models.

** Updated version of this model available on SRCMOD website is used in this work.

Supplementary Table 2 | Rupture models of the Tohoku-oki earthquake obtained without using seafloor GPS data

Model No.	Rupture model	Data used	Peak slip* (m)	Peak slip along the corridor (m)
25	Ammon et al., 2011 (27)	Teleseismic P wave, Rayleigh wave, high-rate GPS	40.0	35.4
26	Bletery et al., 2014 (28)**	High-rate GPS, strong motion, teleseismic waves, tsunami, land GPS	64.0	48.4
27	Diao et al., 2013 (29)	Land GPS	45.8	44.2
28	Frankel et al., 2013 (30)	Strong motion and High-rate GPS	65.6	62.1
29	Fujii et al., 2011 (31)	Tsunami (coastal tide gauge, offshore GPS wave, pressure gauge, open ocean buoy)	47.9	41.9
30	Hayes, 2011 (32)	Teleseismic body and surface waves	33.5	31.9
31	Ide et al., 2011 (33)	Teleseismic waves (empirical Green's function)	30.7	23.4
32	Lay et al., 2011 (34)	Teleseismic P wave	58.1	55.6
33	Maeda et al., 2011 (35)	Tsunami (coastal tide gauge, seafloor pressure gauge)	57.0	57.1
34	Maercklin et al., 2012 (36)	Accelerometer (strong motion) (back projection)	54.8	29.5
35	Melgar and Bock, 2015 (37)	High-rate GPS, strong motion, tsunami (wave gauge)	61.7	55.7
36	Miyazaki et al., 2011 (38)	Land GPS	34.2	33.0
37	Ozawa and Fujita, 2013 (39)	InSAR, land GPS	28.7	28.4
38	Saito et al., 2011 (40)	Tsunami (pressure gauge, GPS wave gauge)	33.4	24.0
39	Satake et al., 2013 (41)	Tsunami (open ocean buoy, coastal tide gauge, pressure gauge)	69.1	58.9

40	Satriano et al., 2014 (42)	Teleseismic P wave (back projection technique)	56.5	52.9
41	Simons et al., 2011 (43)	Land GPS, tsunami (open ocean buoy)	59.7	38.0
42	Suzuki et al., 2011 (44)	Low frequency (0.01–0.125 Hz) strong motion	48.3	45.5
43	Yagi and Fukahata, 2011 (45)	Teleseismic P wave	51.2	45.6
44	Yamazaki et al., 2011 (46)	Teleseismic P wave, Tsunami (GPS buoy, wave gauges, open ocean Buoy)	70.3	66.7
45	Yoshida et al., 2011 (47)	Strong motion	46.9	23.5

* The peak values were obtained from the original finite fault slip models.

** Seafloor GPS data were testing purpose only, not used in the preferred model.

Supplementary Table 3 | SDB models presented in this paper*

SDB Model	ODB to fit	Average slip	Slip gradient**	Depth adjustment***	RMS deviation	Figure Number
Optimal	1999-2011 MY102	62.0	5.0	5.0	8.481	3
Huge slip	1999-2011 MY102	90.0	5.0	0.0	8.948	S4
Slip increase	1999-2011 MY102	62.0	25.0	6.0	8.651	6
Slip decrease	1999-2011 MY102	62.0	-15.0	4.0	8.731	7
2004	2004-2011 MY102	52.0	-12.0	1.1	8.116	S3
North track	1999-2011 MY101	42.0	20.0	0.5	10.589	S5

* Slip, depth adjustment, and RMS deviation are all in meters.
** Slip gradient is given as linear change (m) over the most near-trench 40 km. Positive values indicate increase towards the trench.
*** Given the average slip and slip gradient in each model, the listed depth adjustment is the optimal value (for obtaining the lowest RMS deviation).

Supplementary references

1. Ozawa, S. *et al.* Preceding, coseismic, and postseismic slips of the 2011 Tohoku earthquake, Japan. *J. Geophys. Res.* **117**, B07404 (2012). doi:10.1029/2011JB009120
2. Sato, M. *et al.* Displacement above the hypocenter of the 2011 Tohoku-Oki earthquake. *Science* **332**, 1395 (2011). doi:10.1126/science.1207401
3. Kido, M., Osada, Y., Fujimoto, H., Hino, R. & Ito, Y. Trenchnormal variation in observed seafloor displacements associated with the 2011 Tohoku-Oki earthquake. *Geophys. Res. Lett.* **38**, L24303 (2011). doi:10.1029/2011GL050057
4. Ito, Y. *et al.* Frontal wedge deformation near the source region of the 2011 Tohoku-Oki earthquake. *Geophys. Res. Lett.* **38**, L00G05 (2011). doi:10.1029/2011GL048355
5. Gusman, A. R., Tanioka, Y., Sakai, S. & Tsushima, H. Source model of the great 2011 Tohoku earthquake estimated from tsunami waveforms and crustal deformation data. *Earth Planet. Sci. Lett*. **341-344**, 234-242 (2012). doi:10.1016/j.epsl.2012.06.006
6. Hooper, A. *et al.* Importance of horizontal seafloor motion on tsunami height for the 2011 Mw=9.0 Tohoku-Oki earthquake. *Earth Planet. Sci. Lett*. **361**, 469-479 (2013). doi:10.1016/j.epsl.2012.11.013
7. Iinuma, T. *et al.* Coseismic slip distribution of the 2011 off the Pacific Coast of Tohoku Earthquake (M9.0) refined by means of seafloor geodetic data. *J. Geophys. Res.* **117**, B07409 (2012). doi:10.1029/2012JB009186
8. Imakiire, T. & Koarai, M. Wide-area land subsidence caused by "the 2011 off the Pacific Coast of Tohoku Earthquake". *Soils and Foundation* **52(5)**, 842-855 (2012). doi:10.1016/j.sandf.2012.11.007
9. Ito, T., Ozawa, K., Watanabe, T. & Sagiya, T. Slip distribution of the 2011 off the Pacific coast of Tohoku Earthquake inferred from geodetic data. *Earth Planets Space* **63(7)**, 627–630 (2011). doi:10.5047/eps.2011.06.023
10. Kubo, H. & Kakehi, Y. Source process of the 2011 Tohoku earthquake estimated from the joint inversion of teleseismic body waves and geodetic data including seafloor observation data: Source model with enhanced reliability by using objectively determined inversion settings. *Bull. Seismol. Soc. Am.* **103**, 1195-1220 (2013). doi: 10.1785/0120120113
11. Kyriakopoulos, C., Masterlark, T., Stramondo, S., Chini, M. & Bignami, C. Coseismic slip distribution for the Mw 9 2011 Tohoku-Oki earthquake derived from 3-D FE modeling. *J.*

Geophys. Res. Solid Earth **118**, 3837-3847 (2013). doi:10.1002/jgrb.50265
12. Lee, S.-J., Huang, B.-S., Ando, M., Chiu, H.-C. & Wang, J.-H. Evidence of large scale repeating slip during the 2011 Tohoku-Oki earthquake. *Geophys. Res. Lett.* **38**, L19306 (2011), doi:10.1029/2011GL049580
13. Minson, S. E. *et al.* Bayesian inversion for finite fault earthquake source models-II: the 2011 great Tohoku-oki, Japan earthquake. *Geophys. J. Int.* **198**, 922-940 (2014). doi:10.1093/gji/ggu170
14. Perfettini, H. & Avouac, J. P. The seismic cycle in the area of the 2011 Mw 9.0 Tohoku-Oki earthquake. *J. Geophys. Res. Solid Earth* **119**, 4469-4515 (2014). doi:10.1002/2013JB010697
15. Pollitz, F. F., Bürgmann, R. & Banerjee, P. Geodetic slip model of the 2011 M9.0 Tohoku earthquake. *Geophys. Res. Lett.* **38**, L00G08 (2011). doi:10.1029/2011GL048632
16. Pulvirenti, F., Jin, S. & Aloisi, M. An adjoint-based FEM optimization of coseismic displacements following the 2011 Tohoku earthquake: new insights for the limits of the upper plate rebound. *Phys. Earth Planetary Interiors* **237**, 25-39 (2014). doi:10.1016/j.pepi.2014.09.003
17. Romano, F. *et al.* Structural control on the Tohoku earthquake rupture process investigated by 3D FEM, tsunami and geodetic data. *Sci. Rep.* **4**, 5631 (2014). doi:10.1038/srep05631
18. Shao, G., Chen, J. & Archuleta, R. Quality of earthquake source models constrained by teleseismic waves: Using the 2011 M9 Tohoku-oki earthquake as an example. (Poster 93, presented at Incorporated Research Institutions for Seismology Workshop, Boise, Idaho, 13-15 June) (2012); available at http://www.iris.edu/hq/iris_workshop2012/scihi/WebPages/0115.html.
19. Silverii, F., Cheloni, D., D'Agostino, N., Selvaggi, G. & Boschi E. Post-seismic slip of the 2011 Tohoku-Oki earthquake from GPS observations: Implications for depth-dependent properties of subduction megathrusts. *Geophys. J. Int.* **198(1)**, 580-596 (2014). doi:10.1093/gji/ggu149
20. Wang, C., Ding, X., Shan, X., Zhang, L. & Jiang, M. Slip distribution of the 2011 Tohoku earthquake derived from joint inversion of GPS, InSAR and seafloor GPS/acoustic measurements. *J. Asian Earth Sci.* **57**, 128-136 (2012). doi:10.1016/j.jseaes.2012.06.019
21. Wang, R. *et al.* The 2011 Mw 9.0 Tohoku Earthquake: Comparison of GPS and Strong

Motion Data. *Bull. Seismol. Soc. Am.* **103**, 1336-1347 (2013). doi:10.1785/0120110264

22. Wei, S., Graves, R., Helmberger, D., Avouac, J. & Jiang, J. Sources of shaking and flooding during the Tohoku-oki earthquake: A mixture of rupture styles. *Earth Planet. Sci. Lett.* **333-334**, 91-100 (2012). doi:10.1016/j.epsl.2012.04.006
23. Wei, Y. *et al.* Tsunami forecast by joint inversion of real-time tsunami waveforms and seismic or GPS data: application to the Tohoku 2011 tsunami. *Pure Appl. Geophys.* **171**, 3281-3305 (2014). doi:10.1007/s00024-014-0777-z
24. Yokota, Y. *et al.* Joint inversion of strong motion, teleseismic, geodetic, and tsunami datasets for the rupture process of the 2011 Tohoku earthquake. *Geophys. Res. Lett.* **38**, L00G21 (2011). doi:10.1029/2011GL050098
25. Yue, H. & Lay, T. Source rupture models for the Mw 9.0 2011 Tohoku earthquake from joint inversions of high-rate geodetic and seismic data. *Bull. Seismol. Soc. Am.* **103(2b)**, 1242-1255 (2013). doi:10.1785/0120120119
26. Zhou, X., Cambiotti, G., Sun, W. & Sabadini, R. The coseismic slip distribution of a shallow subduction fault constrained by prior information: the example of 2011 Tohoku (Mw 9.0) megathrust earthquake. *Geophys. J. Int.* **199(2)**, 981-995 (2014). doi: 10.1093/gji/ggu310
27. Ammon, C. J., Lay, T., Kanamori, H. & Cleveland, M. A rupture model of the 2011 off the Pacific coast of Tohoku Earthquake. *Earth Planets Space* **63**, 693-696 (2011). doi:10.5047/eps.2011.05.015
28. Bletery, Q. *et al.* A detailed source model for the Mw 9.0 Tohoku-Oki earthquake reconciling geodesy, seismology, and tsunami records. *J. Geophys. Res. Solid Earth* **119**, 7636-7653 (2014). doi:10.1002/2014JB011261
29. Diao, F. *et al.* Overlapping post-seismic deformation processes: afterslip and viscoelastic relaxation following the 2011 Mw 9.0 Tohoku (Japan) earthquake. *Geophys. J. Int.* **196(1)**, 218-229 (2014). doi:10.1093/gji/ggt376
30. Frankel, A. Rupture history of the 2011 M 9 Tohoku Japan earthquake determined from strong-motion and high-rate GPS recordings: subevents radiating energy in different frequency bands. *Bull. Seismol. Soc. Am.* **103**, 1290-1306 (2013). doi:10.1785/0120120148
31. Fujii, Y., Satake, K., Sakai, S., Shinohara, M. & Kanazawa, T. Tsunami source of the 2011 off the Pacific coast of Tohoku Earthquake. *Earth Planets Space* **63**, 815-820 (2011). doi:10.5047/eps.2011.06.010

32. Hayes, G. Rapid source characterization of the 03-11-2011 Mw 9.0 off the Pacific coast of Tohoku Earthquake. *Earth Planets Space* **63**, 529-534 (2011). doi:10.5047/eps.2011.05.012
33. Ide, S., Baltary, A. & Beroza, G. C. Shallow dynamic overshoot and energetic deep rupture in the 2011 M_w 9.0 Tohoku-Oki Earthquake. *Science* **332**, 1425-1429 (2011). doi:10.1126/science.1207020
34. Lay, T., Ammon, C. J., Kanamori, H., Xue, L. & Kim, M. J. Possible large near-trench slip during the 2011 M_w 9.0 off the Pacific coast of Tohoku earthquake. *Earth Planets Space* **63**, 687-692 (2011). doi: 10.5047/eps.2011.05.033
35. Maeda, T., Furumura, T., Sakai, S. & Shinohara, M. Significant tsunami observed at ocean-bottom pressure gauges during the 2011 off the Pacific coast of Tohoku Earthquake. *Earth Planets Space* **63**, 803-808 (2011). doi:10.5047/eps.2011.06.005
36. Maercklin, N., Festa, G., Colombelli, S. & Zollo, A. Twin ruptures grew to build up the giant 2011 Tohoku, Japan, earthquake. *Sci. Rep.* **2**, 709 (2012). doi:10.1038/srep00709
37. Melgar, D. & Bock, Y. Kinematic earthquake source inversion and tsunami runup prediction with regional geophysical data. *J. Geophys. Res. Solid Earth* **120**, 3324-3349 (2015). doi:10.1002/2014JB011832
38. Miyazaki, S., McGuire, J. J. & Segall, P. Seismic and aseismic fault slip before and during the 2011 off the Pacific coast of Tohoku earthquake. *Earth Planets Space* **63(7)**, 637-642 (2011). doi:10.5047/eps.2011.07.001
39. Ozawa, T. & Fujita, E. Local deformations around volcanoes associated with the 2011 off the Pacific coast of Tohoku earthquake. *J. Geophys. Res. Solid Earth* **118**, 390-405 (2013). doi:10.1029/2011JB009129
40. Saito, T., Ito, Y., Inazu, D. & Hino, R. Tsunami source of the 2011 Tohoku-Oki earthquake, Japan: Inversion analysis based on dispersive tsunami simulations. *Geophys. Res. Lett.* **38**, L00G19 (2011). doi:10.1029/2011GL049089
41. Satake, K., Fujii, Y., Harada, T. & Namegaya, Y. Time and space distribution of coseismic slip of the 2011 Tohoku earthquake inferred from tsunami waveform data. *Bull. Seismol. Soc. Am.* **103(2B)**, 1473-1492 (2013). doi:10.1785/0120120122
42. Satriano, C. *et al.* Structural and thermal control of seismic activity and megathrust rupture dynamics in subduction zones: Lessons from the M_w 9.0, 2011 Tohoku earthquake. *Earth Planet. Sci. Lett.* **403**, 287-298 (2014). doi.org/10.1016/j.epsl.2014.06.037
43. Simons, M. *et al.* The 2011 magnitude 9.0 Tohoku-Oki earthquake: Mosaicking the megathrust from seconds to centuries. *Science* **332**, 1421-1425 (2011). doi:10.1126/science.1206731

44. Suzuki, W., Aoi, S., Sekiguchi, H. & Kunugi, T. Rupture process of the 2011 Tohoku-Oki megathrust earthquake (M9.0) inverted from strong motion data. *Geophys. Res. Lett.* **38**, L00G16 (2011). doi:10.1029/2011GL049136
45. Yagi, Y. & Fukahata, Y. Rupture process of the 2011 Tohoku-oki earthquake and absolute elastic strain release. *Geophys. Res. Lett.* **38**, L19307 (2011). doi:10.1029/2011GL048701
46. Yamazaki, Y., Lay, T., Cheung, K. F., Yue, H. & Kanamori, H. Modeling near-field tsunami observations to improve finite-fault slip models for the 11 March 2011 Tohoku earthquake. *Geophys. Res. Lett.* **38**, L00G15 (2011). doi:10.1029/2011GL049130
47. Yoshida, Y., Ueno, H., Muto, D. & Aoki, S. Source process of the 2011 Off the Pacific Coast of Tohoku Earthquake with the combination of teleseismic and strong motion data. *Earth Planets Space* **63**, 565-568 (2011). doi:10.5047/eps.2011.05.011
